# Treatment and prognostic factors of pituicytoma: a single-center experience and comprehensive literature review

**DOI:** 10.1007/s11102-021-01152-5

**Published:** 2021-05-12

**Authors:** Liu-Dong Wei, Chao Li, Da Li, Xing-Ju Liu, Run-Ting Li, Lian-Wang Li, Jun-Mei Wang, Da-Biao Zhou

**Affiliations:** 1 Department of Neurosurgery, Beijing Tiantan Hospital, Capital Medical University, No.119 South Fourth Ring West Road, Fengtai District, Beijing, 100070 People’s Republic of China; 2 Department of Neuropathology, Beijing Neurosurgical Institute, No.119 South Fourth Ring West Road, Fengtai District, Beijing, 100070 People’s Republic of China

**Keywords:** Diagnosis, Oncology, Pituicytoma, Radiotherapy, Total resection, Treatment strategy

## Abstract

**Purpose:**

Preoperative diagnosis of pituicytomas is difficult, and management and prognostic factors remain ambiguous. The purpose of this study was to elucidate the radiological characteristics of pituicytoma, to assess the risk factors affecting tumor progression, and to propose the optimal treatment regimen based on comprehensive analysis.

**Methods:**

We reviewed the clinical data of 22 patients with pituicytoma confirmed pathologically in our institution. In addition, 93 cases of pituicytoma in the previous literature were recruited. The individual data of 115 patients were analyzed to evaluate the adverse factors affecting pituicytoma progression.

**Results:**

In the combined cohort, 3 of 61 patients who underwent gross-total resection (GTR) developed recurrence (4.9%); of the 54 patients who received non-GTR, 19 progressed (35.2%). Univariate and multivariate Cox regression analysis verified male gender (HR 2.855, 95% CI 1.008–8.089; p = 0.048), TS (transsphenoidal surgery; HR 3.559, 95% CI 1.015–12.476; p = 0.047), and non-GTR (HR 4.388, 95%CI 1.240–15.521; p = 0.022) were independent unfavorable factors for pituicytoma progression. A multivariate logistic regression model verified that tumor diameter ≥ 1.85 cm (OR 4.859, 95% CI 1.335–17.691; p = 0.016) was independent adverse factors for GTR. Compared with TS, OT (open transcranial) is more likely to have postoperative complications (OR 3.185, 95% CI 1.020–9.944; p = 0.046), especially vision deterioration (OR 37.267, 95% CI 4.486–309.595; p = 0.001).

**Conclusion:**

Based on our findings, GTR was advocated as an optimal treatment for pituicytomas. However, in order to avoid damage to important structures, partial resection is acceptable. After that, adjuvant radiotherapy is recommended for male patients with high Ki-67 index, and the remaining patients can be followed up closely. When the tumor recurs or progresses, it is recommended to re-operate and remove the lesion completely as far as possible. If GTR is still not possible, postoperative radiotherapy for the residual tumor is recommended.

**Supplementary Information:**

The online version contains supplementary material available at 10.1007/s11102-021-01152-5.

## Introduction

Pituicytomas are exceedingly rare non-neuroendocrine neoplasms in the sellar or suprasellar region, originating from the neurohypophysis or infundibulum [1]. The tumors are histologically benign, but their ample vascular characteristics make complete resection difficult, and local recurrence or progression following partial resection is not uncommon [2–5]. Management of pituicytoma remains a controversial issue due to its rarity and lack of a large series of cases describing the adverse factors for tumor progression. A recent study conducted by Ogiwara et al. showed that incomplete resection is a risk factor for tumor recurrence [6]. However, no specific treatment strategy has been proposed and the applicability of radiotherapy is still unclear. Single case reports of successful treatment and good prognosis of pituicytomas are not so convincing, and generalization to a larger number of patients is limited. Therefore, we retrospectively reviewed the clinical data of all patients with pathologically confirmed pituicytoma in our institution, and extensively reviewed the previously published literature. Our aim is to assess the risk factors for pituicytoma progression based on data collected from our cases and previous literature, and then to propose the optimal treatment algorithm.

## Materials and methods

### Patient population

Institutional review board approval was obtained for this project. All patients with pathologically confirmed pituicytomas treated at our hospital in the period from 2010 to 2020 were eligible for inclusion into the present study. Patient data were extracted where available, including age, sex, symptoms, histopathological analysis, surgical approach and outcome, use of radiation therapy, tumor characteristics (size, volume, location and consistency), and follow-up data. Endocrine examination includes morning fasting cortisol, adrenocorticotropic hormone (ACTH), thyroid function test (thyroid-stimulating hormone (TSH), total triiodothyronine (TT3), total thyroxine (TT4), free T3 (FT3), and free T4 (FT4)), follicle-stimulating hormone (FSH), luteinizing hormone (LH), progesterone (P4), growth hormone (GH), insulin-like growth factor-1(IGF-1), and prolactin (PRL).

Tumor volumes were calculated using the cubature formula: volume = (a × b × c)/2,where a, b, and c represent the maximum diameters measured on the axial, sagittal and coronal magnetic resonance imaging (MRI) scans respectively. The texture of tumor is defined semi-quantitatively and divided into three subtypes: (1) soft, which can be aspirated by aspirator; (2) fibrous, which can’t be aspirated, but can be cut with scissors; (3) hard, which can’t be cut by scissors. The extent of tumor removal was determined by comparison of pre- and postoperative volumetric analysis of T1-weighted contrast-enhanced MRI scans, which was recorded as gross-total resection (GTR; no residue) or non-GTR (residue was found on postoperative contrast-MRI scans before discharge). Clinical and radiological follow-up was conducted every 6 months for the first 2 years, followed by annual outpatient or telephone follow-up.

### Pathological examination

All specimens were fixed by 4% neutral formalin, dehydrated routinely, paraffin-embedded, and prepared into 4‐μm sections, which were then stained with hematoxylin–eosin (HE). The immunohistochemical staining was implemented for thyroid transcription factor‐1 (TTF‐1), S‐100, vimentin, glial fibrillary acidic protein (GFAP), epithelial membrane antigen (EMA), synaptophysin, and Ki-67. Diagnosis was established based on the typical histopathological features of pituicytoma.

### Pooled analysis of prior published cases

Using the Embase, PubMed and Cochrane databases, two independent authors reviewed English-language pituicytoma literature published between January 1958 and May 2020.The keyword used in the search was “pituicytoma”. Inclusion criteria: (1) pathological diagnosis of pituicytoma; (2) detailed information of treatment, neurological outcome, status of recurrence or death. Exclusion criteria: (1) follow-up data or extent of resection was unavailable; (2) basic research, not clinical report, no clinical data for statistical analysis (3) pathological diagnosis was undefined; (4) duplicated report. Histological images of the tumor and all written descriptions available for pathological diagnosis were reviewed by a board certified neuropathologist (J.M.W.) in accordance with the latest World Health Organization (WHO) diagnostic criteria [7, 8]. The details of each patient from our institution and previous literature were summarized in Supplementary Tables 1, 2, and 3 respectively.

**Fig. 1:**
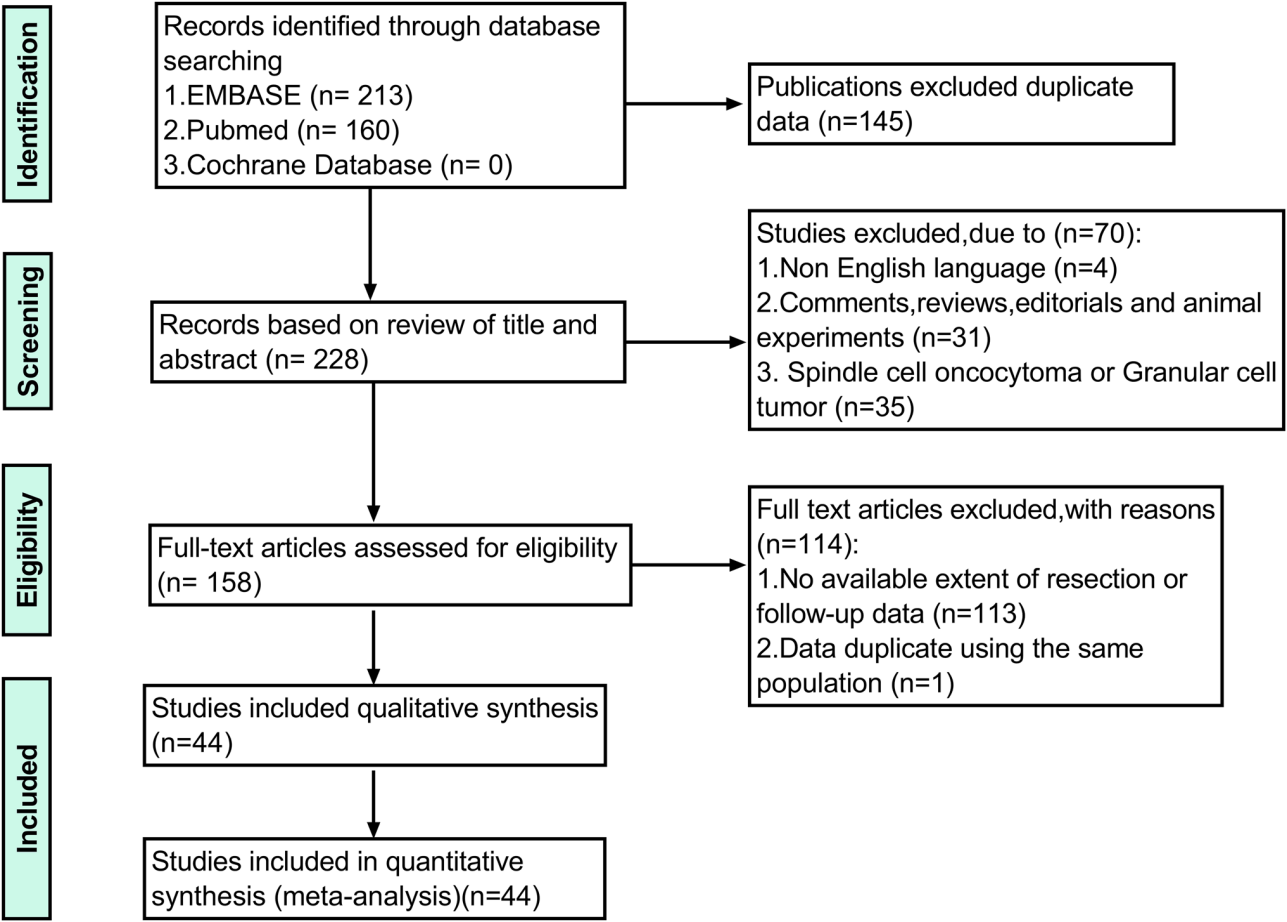
The PRISMA flowchart shows the inclusion and exclusion processes used for analysis

Previously published data were processed and used in accordance with the PRISMA (Preferred Reporting Items for Systematic Reviews and Meta-Analyses) guidelines. The process is as follows in Fig. 1. Two review authors independently screened the search results. The authors’ differences on bias in specific studies are resolved through discussion, with the participation of a third review author if necessary. This study was registered with the PROSPERO database (http://www.crd.york.ac.uk/prospero/), and its registration No. is CRD42020193499.

### Statistical analysis

Univariate analysis and multivariate Cox regression analysis were used to evaluate the risk factors of progression in patients with pituicytoma. Since there were no deaths during the follow-up period, risk factors for overall survival were not be calculated. Kaplan–Meier method was used to illustrate the survival curve of patients with significant risk factors. The estimated median time to progression (TTP) was not given because the censored cases in some subgroups were more than 50%; therefore, we used the estimated mean TTP (EMTTP) to describe TTP. Log rank (Mantel-Cox) test was used to calculate chi-square values in Kaplan–Meier curves analysis. Univariate logistic regression was used to analyze the influencing factors of GTR and postoperative complications. The cutoff value for tumor diameter of 1.85 cm was determined by receiver operator characteristic (ROC) curve analysis. All analyses were performed by SPSS Statistical Package software (version 24.0, IBM Corp.) with significance set at p < 0.05.

## Results

### Patient demographics and presentations

Over the past 10 years 22 consecutive patients have been operated on in our institution. Among them 11 are males and 11 are females. The age of patients ranges from 24 to 65 years (median 49 years). The median duration of symptoms was 6.0 months (range 1.0–60.0 months), and the most frequent preoperative symptoms were visual disturbance (14 cases), followed by vertigo (9 cases) and headache (8 cases). Endocrine examination showed that 12 patients (54.5%) had normal hormone, 10 patients (45.5%) had abnormal hormone, and all of them showed partial hypopituitarism. Except for 2 patients who had been operated on previously, 20 patients with initial diagnosis were not accurate, including 8 cases of pituitary adenoma, 7 cases of craniopharyngioma, 3 cases of meningioma, 1 case of granular cell tumor and 1 case of hypophysitis (Table 1).

**Table 1:** Clinical features and demographics of patients with pituicytomas from our series and literature

Variable	Current series (n = 22)	Prior studies (n = 93)		Overall (n = 115)	
	Value	No. of available cases	Value	No. of available cases	Value
*Sex*		93		115	
Male	11 (50%)		49 (52.7%)		60 (52.2%)
Female	11 (50%)		44 (47.3%)		55 (47.8%)
*Age, year*		93		115	
Median (range)	49 (24–65)		48 (7–83)		48 (7–83)
Mean ± SD	47.8 ± 9.6		47.8 ± 15.6		48.0 ± 14.6
Main complaint		91		113	
Visual	14 (63.6%)		49 (53.8%)		63 (55.8%)
Headache	8 (36.4%)		33 (36.3%)		41 (36.3%)
Decreased libido	3 (13.6%)		13 (14.3%)		16 (14.2%)
Vertigo	9 (40.9%)		4 (4.4%)		13 (11.5%)
Menstruation abnormality	1 (4.5%)		8 (8.8%)		9 (8.0%)
Polydips; Polyuria	1 (4.5%)		6 (6.6%)		7 (6.2%)
Endocrine disturbance	10 (45.5%)		59 (64.8%)		69 (61.1%)
*Surgical Methods*		71		93	
TS	9 (40.9%)		52 (73.2%)		61 (65.6%)
OT	13 (59.1%)		19 (26.8%)		32 (34.4%)
*Extent of removal*		93		115	
Non-GTR	15 (68.2%)		39 (41.9%)		54 (47.0%)
GTR	7 (31.8%)		54 (58.1%)		61 (53.0%)
Complications^a^		55		75	
Visual worsening	9 (45.0%)		5 (9.1%)		14 (18.7%)
Diabetes insipidus	12 (60.0%)		19 (34.5%)		31 (41.3%)
Hypopituitarism	18 (90.0%)		22 (40.0%)		40 (53.3%)
No complications	1 (5.0%)		22 (40.0%)		23 (30.7%)
*Follow-up duration, month*		93		115	
Median (range)	22 (5–86)		24 (3–132)		24 (3–132)
Mean ± SD	31.3 ± 24.9		31.4 ± 28.5		31.3 ± 27.7
Recurrence/progress	6 (27.3%)	93	16 (17.2%)	115	22 (19.1%)
Death during follow up	0	93	0	115	0

*TS* transsphenoidal surgery; *OT* open transcranial; *GTR* gross-total resection; *SD* standard deviation

^a^Two patients who had previously undergone surgery at other hospitals were excluded because complications could not be obtained. The chief complaint, the surgical methods, and the extent of removal were obtained by inquiring the patient, consulting the medical records, and comparing the MRI before and after the surgery respectively. All of these were the data of the patient’s first visit

### Surgical findings

Microscopically, the tumors were grayish-red (9 cases), grayish-yellow (5 cases), grayish-white (4 cases), red (3 cases), and grayish-brown (1 case). The texture was hard in 1 case, fibrous in 9 cases, mixed soft- fibrous in 3 cases and soft in 9 cases. There were 6, 8, 5, and 3 tumors with extremely rich, abundant, moderate, and mild vascularization, respectively. Remarkably, the surgical records of 9 (40.9%) patients specifically stated that the tumor adhered closely to the adjacent structures and could not be forcibly separated. The intraoperative blood loss ranged from 100 to 12000 ml, the average blood loss was 879.55 ml, and the median was 250 ml (Supplemental table 2, for further details).

### Surgical outcomes

Of the 22 patients, 13 underwent open transcranial surgeries, 5 underwent endoscopic transnasal transsphenoidal surgeries and 4 underwent microscopic transnasal transsphenoidal surgeries. Based on postoperative MRI scans, GTR was achieved in 7 (31.8%) surgeries and non-GTR in 15 (68.2%) surgeries. The main postoperative complications were hypopituitarism in 18 cases (90.0%), diabetes insipidus in 12 cases (60%), and decreased vision in 9 cases (45%). After a median follow-up of 22 months (range 5–86 months), 6 patients with non-GTR showed residual tumor progression (Table 1).

### Individual patient data of previous cases

A total of 93 patients were collected, including 49 males and 44 females. The age of patients ranges from 7 to 83 years (median 48 years). The most frequent clinical complaint was visual disorders (n = 49; 53.8%), followed by headache (n = 33; 36.3%). Endocrine examination showed that 59 (64.8%) people had abnormal endocrine, of whom 35 patients had partial or total hypopituitarism.

Of the 93 patients, 54 (58.1%) achieved GTR and 39 (41.9%) non-GTR. Data related to postoperative complications was documented in 55 patients; the most common were hypopituitarism (n = 22; 40.0%), DI (n = 19; 34.5%) and visual worsening (n = 5; 9.1%). No surgical complications were reported in 22 (40.0%) cases. After a mean follow-up of 31.4 months (range 3–132 months), 3 patients with GTR had recurrence and 13 patients with non-GTR had residual tumor progression, with no death (Table 1).

### Radiographic characteristics

In terms of imaging studies, 112 cases could obtain the topographical description of tumors; 48 (42.9%) were intrasellar with suprasellar extension, 36 (32.1%) were suprasellar, and 28 (25.0%) exclusively intrasellar. Their consistency (n = 80) was predominantly solid (n = 66; 82.5%), with a few cystic changes (n = 14; 17.5%). The major diameter of tumors (n = 74) ranged from 2 to 55 mm (median: 20). The mean tumor volume (n = 52) was 4.78 cm^3^ (median: 3.08; range 0.019–31.94 cm^3^).

**Fig. 2:**
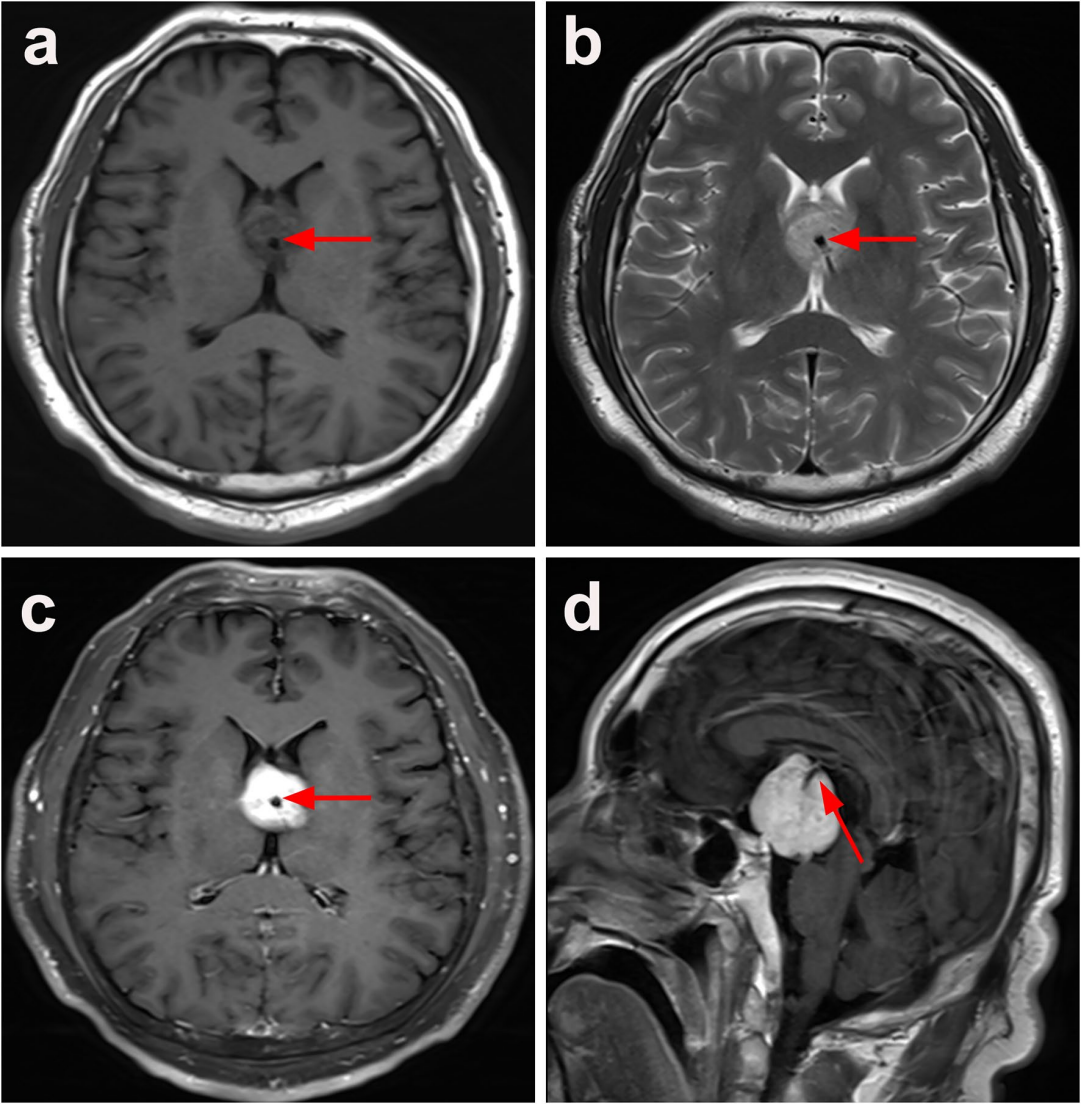
Brain magnetic resonance imaging showing flow void within the tumor. The mass mainly shows isointensity on axial T1WI (**a**) and T2WI (**b**); Postcontrast axial (**c**) and saggital (**d**) T1WI show a suprasellar lesion with a well-defined margins and obvious homogeneous enhancement. Note that within the mass there is a flow void (arrows in **a–d**) demonstrating the strong arterial supply of a highly vascularized tumor

In the combined cohort, only 40 patients provided relatively detailed MRI descriptions, including T1-weighted images (T1WI), T2-weighted images (T2WI), and contrast-enhanced images. Among them, 14 patients (35%) exhibited intermediate signal intensity on T1WI and T2WI, 10 patients (25%) showed isointense on T1WI and hyperintense on T2WI, and 31 patients (77.5%) presented homogeneous enhancement. When studied separately, the most common manifestations were isointensity on T1WI (n = 29, 72.5%) and hyperintensity on T2WI (n = 18, 45%). Notably, significant intratumoral flow voids were observed in 5 patients from our hospital (Fig. 2). On CT scans (n = 14), 11 patients (78.6%) demonstrated isodense and 3 patients (21.4%) showed hyperdense, without calcification (Table 2).

**Table 2:** Radiological and pathological features of pituicytomas in the combined data

Variable	Numerical value
*Location*	112
Intrasellar	28 (25.0%)
Suprasellar	36 (32.1%)
Intrasellar + Suprasellar	48 (42.9%)
*Consistency*	80
Solid	66 (82.5%)
Solid + Cystic	14 (17.5%)
*Major Diameter (mm)*	74
Median (range)	20 (2–55)
Mean ± SD	20.6 ± 9.8
*Lesion volume in cm*^*3*^	52
Median (range)	3.08 (0.019–31.94)
Mean ± SD	4.78 ± 6.55
*MRI feature*	40
*T1WI*	
Isointense	29 (72.5%)
Hypointense	8 (20%)
Isointense to hypointense	2 (5%)
Isointense to hyperintense	1 (2.5%)
*T2WI*	
Hyperintense	18 (45%)
Isointense	14 (35%)
Isointense to hyperintense	4 (10%)
Isointense to hypointense	3 (7.5%)
Hypointense	1 (2.5%)
*T1WI&T2WI*	
Isointense T1& isointense T2	14 (35%)
Isointense T1& hyperintense T2	10 (25%)
Hypointense T1& hyperintense T2	7 (17.5%)
Isointense T1& iso- to hyperintense T2	3 (7.5%)
Isointense T1& hypointense T2	1 (2.5%)
Isointense T1& iso- to hypointense T2	1 (2.5%)
Hypointense T1& iso- to hypointense T2	1 (2.5%)
Iso- to hyperintense T1& iso- to hypointense T2	1 (2.5%)
Iso- to hypointense T1& hyperintense T2	1 (2.5%)
Iso- to hypointense T1& iso- to hyperintense T2	1 (2.5%)
*Enhancement*	
Heterogeneous	9 (22.5%)
Homogeneous	31 (77.5%)
*CT feature*	14
Isodense	11 (78.6%)
Hyperdense	3 (21.4%)
No calcification	14 (100%)
*Immunohistochemical*	
TTF-1 positive/negative	51 (100%)/0
S100 positive/negative	85 (97.7%)/2 (2.3%)
Vimentin positive/negative	45 (93.7%)/3 (6.3%)
GFAP positive/negative	57 (69.5%)/25 (30.5%)
EMA positive/negative	24 (38.7%)/38 (61.3%)
Synaptophy positive/negative	11 (33.3%)/22 (66.7%)
Ki-67	53
Median (range)	2% (0.5–15%)
Mean ± SD	3.0% ± 2.9%

*MRI* magnetic resonance imaging; *T1WI* T1-weighted images; *T2WI* T2-weighted images; *CT* computed tomography; *SD* standard deviation; *TTF*‐1 thyroid transcription factor‐1; *GFAP* glial fibrillary acidic protein; *EMA* epithelial membrane antigen

### Histological characteristics

**Fig. 3:**
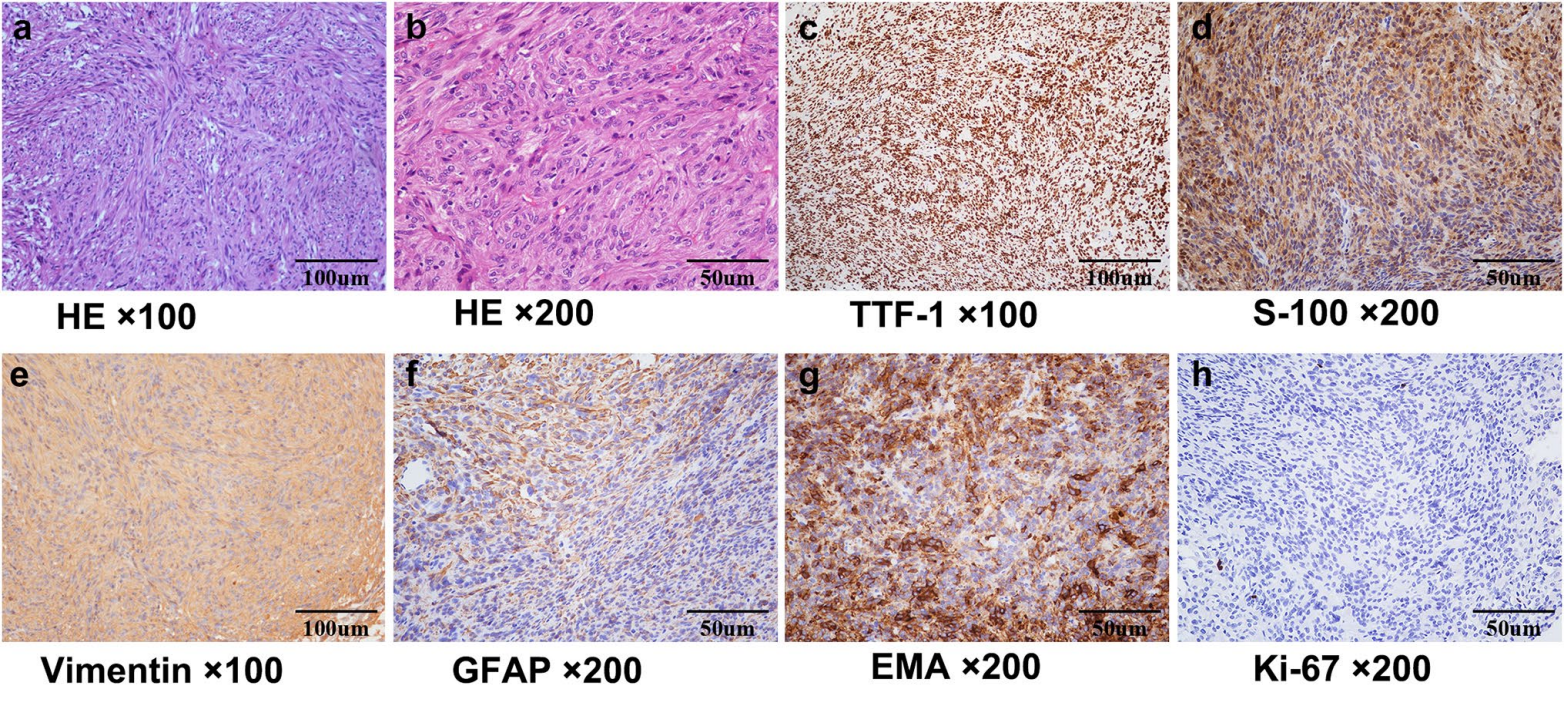
Histopathological examination revealed the typical morphological and immunohistochemical features of pituicytoma. **a** and **b** HE staining showed that the tumor cells were spindle shaped, with unclear boundary, abundant cytoplasm, fine nuclear chromatin and dense interweaving, arranged in fascicles and storiform. **c**–**e** The tumor displayed diffuse immunoreactivity for TTF-1, S-100 protein and vimentin. **f**–**h** The tumor exhibited focal positive staining for GFAP and EMA, with a low Ki67 index. *HE* hematoxylin–eosin; *TTF*‐1 thyroid transcription factor‐1; *GFAP* glial fibrillary acidic protein; *EMA* epithelial membrane antigen

All 22 pituicytomas exhibited similar histological characteristics in our report. HE staining showed that spindle—shaped or oblong cells interlaced into clusters or dense storiform patterns. Tumor cells were eosinophilic with unclear boundaries, abundant cytoplasm and elongated nucleus. Mitotic figures were rare, with occasional observations of myxoid matrix (Fig. 3). Combined with our hospital and previous reports, the results of immunohistochemistry were analyzed as follows: It can be seen that TTF-1, S100 and vimentin were usually positive in pituicytomas, and the positive rates were 100%, 97.7% and 93.7% respectively. The Ki-67 value of tumors ranged from 0.5 to 15% (mean: 3.2%; median: 2%). As shown in the Table 2.

### Factors affecting TTP

**Fig. 4:**
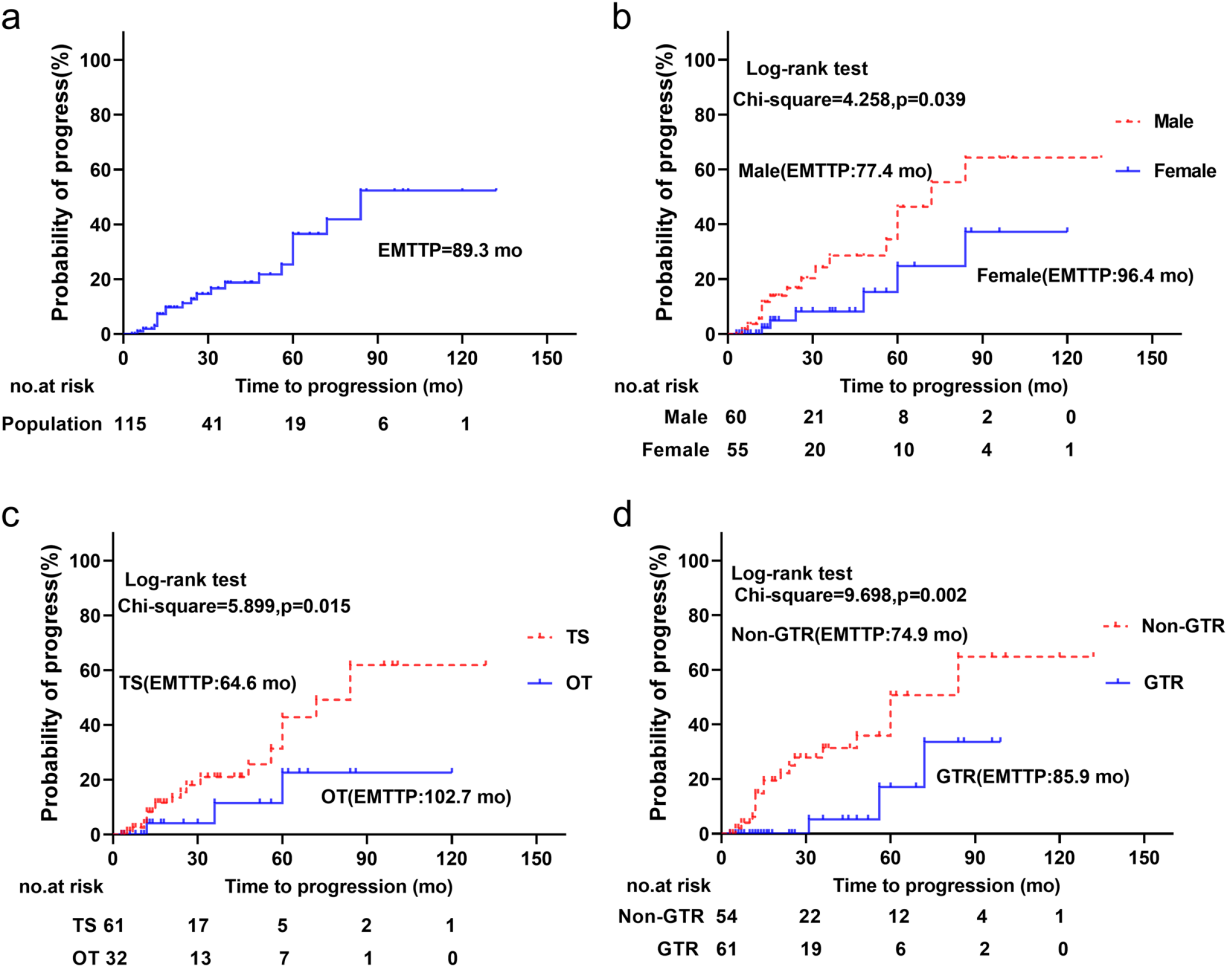
Statistically significant factors for time to progression. **a** TTP in the pooled cohort; **b** male and female groups; **c** TS and OT groups; **d** GTR and non-GTR groups. *TTP* time to progression; *OT* open transcranial; *TS* transsphenoidal surgery; *GTR* gross-total resection; *EMTTP* estimated mean time to progression

**Fig. 5:**
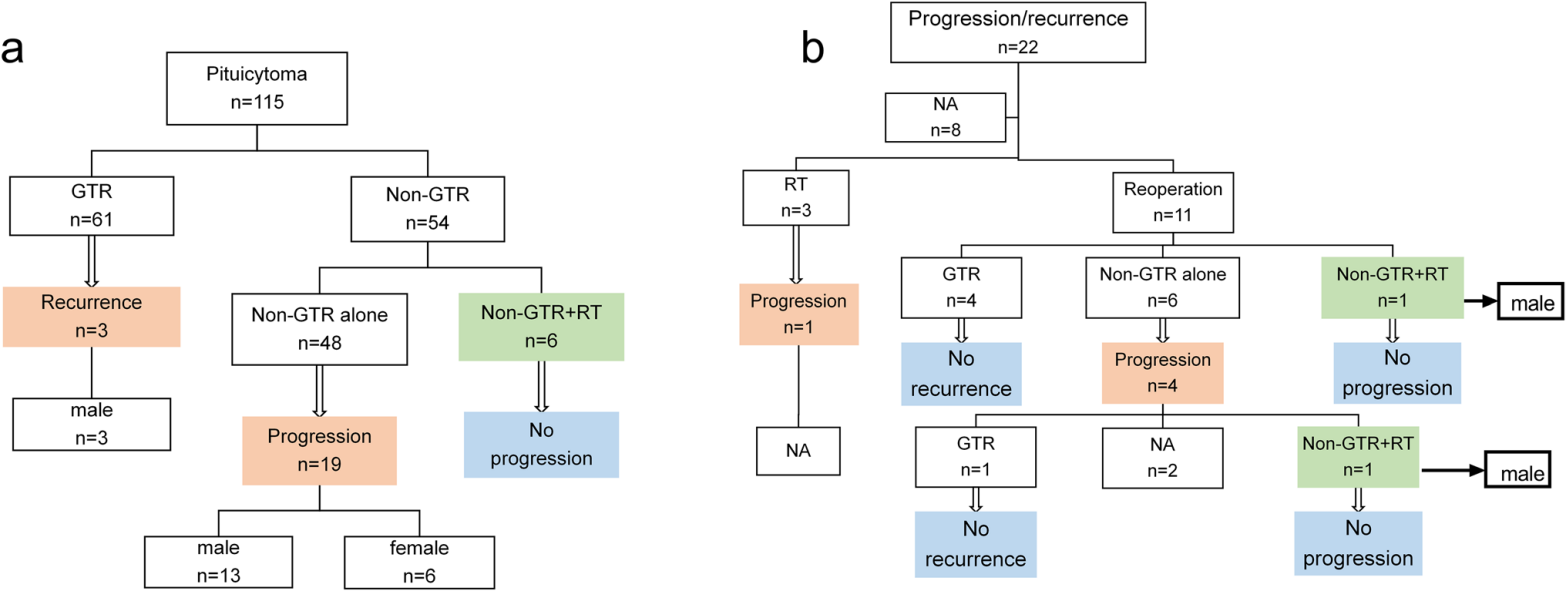
Surgical management data for pituicytomas in the combined cohort. **a** data for patients undergoing initial surgery; **b** Classification of treatment after tumor progression. *GTR* gross-total resection; *RT* radiotherapy; *NA* not available

In the combined cohort, 3 patients relapsed and 19 progressed, with actuarial progression-free rates of 92.8%, 81.2%, and 63.5% at 1, 3, and 5 years, respectively. The estimated mean time to progression was 89.3 months (Fig. 4a). Univariate and multivariate Cox regression analysis verified male gender (HR 2.855, 95% CI 1.008–8.089; p = 0.048) (Fig. 4b), TS (transsphenoidal surgery; HR 3.559, 95% CI 1.015–12.476; p = 0.047) (Fig. 4c), and non-GTR (HR 4.388, 95%CI 1.240–15.521; p = 0.022) (Fig. 4d) were independent unfavorable factors for TTP. Other factors, such as age, tumor size, consistency, location, and Ki-67 were not significant (Table 3). It is worth mentioning that 16 (72.7%) of the 22 patients with recurrence or progression were male, 13 (48.1%) of the 27 male patients with non-GTR developed progression, and all the 3 patients with recurrence after GTR were male (Fig. 5a).

**Table 3:** Univariate and multivariate Cox regression models for predicting risk factors of tumor progression

Variable	No. of Cases	No. of Progression	1-year PFR	3-year PFR	5-year PFR	Univariate Analysis		Multivariate Analysis	
						HR (95% CI)	p Value	HR (95% CI)	p Value
Overall	115	22 (19.1%)	92.8%	81.2%	63.5%				
*Sex*									
Male	60	16 (26.7%)	88.4%	71.5%	53.6%	2.558 (1.000–6.544)	0.039*	2.855 (1.008–8.089)	0.048*
Female	55	6 (10.9%)	97.7%	91.8%	75.3%	1		1	
Age, per 1-year increase	115					1.016 (0.985–1.048)	0.310		
Major diameter, per 1-cm increase	72	10 (13.9%)				1.349 (0.703–2.588)	0.368		
Lesion volume, per 1-cm^3^ increase	50	7 (14%)				1.062 (0.981–1.150)	0.135		
Ki-67, per 1% increase	53	9 (17%)				1.084 (0.900–1.305)	0.397		
*Consistency*									
Solid	66	14 (21.2%)	91.1%	75.5%	63.4%	1.100 (0.246–4.918)	0.901		
Solid + Cystic	14	2 (14.3%)	88.9%	88.9%	44.4%	1			
*Location*									
Intrasellar	28	4 (14.3%)	100%	81.1%	NA	0.465 (0.148–1.460)	0.190		
Suprasellar	36	4 (11.1%)	96.3%	80%	NA	0.685 (0.217–2.160)	0.518		
Intrasellar + Suprasellar	48	12 (25%)	85.7%	82.3%	62.5%	1			
*Surgical methods*									
TS	61	16 (26.2%)	90.3%	73.5%	37.3%	4.100 (1.179–14.262)	0.027*	3.559 (1.015–12.476)	0.047*
OT	32	3 (9.4%)	95.8%	88.5%	77.4%	1		1	
*Radiotherapy*^*a*^									
No RT	109	22 (20.2%)	92.3%	79.8%	60.7%	22.837 (0.23–22,965.274)	0.375		
RT	6	0	100%	100%	100%	1			
*Extent of removal*									
Non-GTR	54	19 (35.2%)	93.8%	68.7%	49.3%	5.509 (1.622–18.711)	0.006*	4.388 (1.240–15.521)	0.022*
GTR	61	3 (4.9%)	100%	94.7%	82.9%	1		1	
*Ki-67*									
Ki-67 < 3%	31	5 (16.1%)	91.5%	91.5%	65.3%	0.852 (0.222–3.276)	0.816		
Ki-67 ≥ 3%	22	4 (18.2%)	100%	64.5%	NA	1			

*PFR* progression free rates; *HR* hazard ratio; *CI* confidence interval; cm, centimeter; *NA* not available; *TS* transsphenoidal surgery; *OT* open transcranial; *GTR* gross-total resection; *RT* radiotherapy

*p < 0.05

^a^Radiotherapy due to residual tumor. All of these were the data of the patient’s first visit

### Factors affecting GTR

A univariate logistic regression analysis showed that tumor size and surgical method were the factors influencing the degree of resection. ROC curve analysis confirmed that the optimal cutoff value for predicting the maximum diameter of GTR was 1.85 cm. A multivariate logistic regression model verified that major diameter ≥ 1.85 cm (OR 4.859, 95% CI 1.335–17.691; p = 0.016) was independent adverse factors for GTR, but surgical method was no longer a significant factor (p = 0.314) (Table 4).

**Table 4:** Univariate and Multivariable Analysis of Predictors of Gross Total Resection Following pituicytoma Surgery

Variables	No. of Patients	No. of GTR	Univariate analysis		Multivariable analysis		
			OR (95% CI)	p Value	No. of Patients	OR (95% CI)	p Value
*Sex*							
Male	60	33 (55%)	1.179 (0.566–2.454)	0.661			
Female	55	28 (50.9%)	1				
Age, per 1-year increase	115		1.015 (0.990–1.042)	0.242			
Major Diameter, per 1-cm increase	72		0.548 (0.319–0.940)	0.029*			
*Major Diameter*							
< 1.85 cm	29	23 (79.3%)	5.863 (1.978–17.38)	0.001*	19	4.859 (1.335–17.691)	0.016*
≥ 1.85 cm	43	17 (39.5%)	1		39	1	
*Consistency*							
Solid + Cystic	14	9 (64.3%)	2.160 (0.653–7.141)	0.207			
Solid	66	30 (45.5%)	1				
*Location*							
Intrasellar	28	16 (57.1%)	1.567 (0.616–4.032)	0.349			
Suprasellar	36	22 (61.1%)	1.857 (0.772–4.470)				
Intrasellar + Suprasellar	48	22 (45.8%)	1				
*Surgical Methods*							
TS	61	35 (57.4%)	2.570 (1.057–6.250)	0.037*	34	1.791 (0.576–5.570)	0.314
OT	32	11 (34.4%)	1		24	1	

*TS* transsphenoidal surgery; *OT* open transcranial; *GTR* gross-total resection

*p < 0.05. All of these were the data of the patient’s first visit

### Factors affecting complications

The factors including gender, age, tumor size, consistency, location, surgical method and degree of resection were used to conduct univariate logistic regression analysis for postoperative complications. The results demonstrated that surgical method was the only factor affecting postoperative complications, and compared with TS, OT(open transcranial) was more prone to occur complications (OR 3.185, 95% CI 1.020–9.944; p = 0.046) (Table 5). Next, we subdivided postoperative complications into hypopituitarism, diabetes insipidus and visual deterioration, and performed statistical analysis in the same way. The results showed that none of the variables had a statistically significant effect on hypopituitarism or diabetes insipidus. In the analysis of the factors affecting visual deterioration, the surgical method was found to be the only influencing factor, and the risk of visual deterioration after OT was significantly higher than that after TS (OR 37.267, 95% CI 4.486–309.595; p = 0.001) (Table 6).

**Table 5:** Univariate analysis of predictors of complications following pituicytoma surgery

Variables	No. of Patients	No. of Incidence of complications	Univariate analysis	
			OR (95% CI)	p Value
*Sex*				
Male	39	29 (74.4%)	1.639 (0.609–4.409)	0.328
Female	36	23 (63.9%)	1	
Age, per 1-year increase	75		1.001 (0.968–1.034)	0.973
Major Diameter, per 1-cm increase	51		0.886 (0.467–1.681)	0.711
*Consistency*				
Solid + Cystic	12	8 (66.7%)	1.100 (0.286–4.228)	0.89
Solid	48	33 (68.8%)	1	
*Location*				
Intrasellar	15	8 (53.3%)	0.468 (0.130–1.676)	0.263
Suprasellar	27	21 (77.8%)	1.432 (0.434–4.723)	
Intrasellar + Suprasellar	31	22 (71.0%)	1	
*Surgical Methods*				
OT	28	23 (82.1%)	3.185 (1.020–9.944)	0.046*
TS	44	26 (59.1%)	1	
*Extent of removal*				
Non-GTR	35	26 (74.3%)	1.556 (0.573–4.221)	0.386
GTR	40	26 (65.0%)	1	

*TS* transsphenoidal surgery; *OT* open transcranial; *GTR* gross-total resection

*p < 0.05. All of these were the data of the patient’s first visit

**Table 6:** Univariate analysis of predictors of visual acuity deterioration following pituicytoma surgery

Variables	No. of Patients	No. of Incidence of postoperative visual acuity deterioration	Univariate analysis	
			OR (95% CI)	p Value
*Sex*				
Male	39	9 (23.1%)	1.860 (0.559–6.194)	0.312
Female	36	5 (13.9%)	1	
Age, per 1-year increase	75		1.008 (0.968–1.050)	0.694
Major Diameter, per 1-cm increase	51		1.216 (0.631–2.342)	0.559
*Consistency*				
Solid + Cystic	12	3 (25%)	1.444 (0.324–6.436)	0.630
Solid	48	9 (18.8%)	1	
*Location*				
Intrasellar	15	1 (6.7%)	0.371 (0.039–3.500)	0.314
Suprasellar	27	7 (25.9%)	1.820 (0.502–6.593)	
Intrasellar + Suprasellar	31	5 (16.1%)	1	
*Surgical Methods*				
OT	28	13 (46.4%)	37.267 (4.486–309.595)	0.001*
TS	44	1 (2.3%)	1	
*Extent of removal*				
Non-GTR	35	9 (25.7%)	2.423 (0.726–8.087)	0.150
GTR	40	5 (12.5%)	1	

*TS* transsphenoidal surgery; *OT* open transcranial; *GTR* gross-total resection

*p < 0.05. All of these were the data of the patient’s first visit

## Discussion

Pituicytoma, previously also known as “choristoma”, “infundibuloma”, “pilocytic astrocytoma” and “granular cell myoblastoma”, was first reported by Scothorne in 1955 and named by Liss and Kahn in [9, 10]. Brat et al. proposed the first pathological diagnostic criteria for this tumor in [11], and it was recognized as a distinct entity in the fourth edition of the WHO Classification of Tumors of the Central Nervous System in [12]. As the tumor is extremely rare, no specific treatment has been proposed for this disease and its prognosis is unknown. On the basis of this series and previous studies, we validated the risk factors affecting the prognosis of pituicytoma for the first time, and proposed corresponding treatment strategies.

### Clinical presentation, radiographic features and diagnosis

In this pooled cohort, the median age was 48 years, suggesting that pituicytomas tend to occur in middle age, and no gender preference was observed. The typical clinical manifestations of pituicytomas are progressive mass effect symptoms, such as abnormal endocrine hormone (61.1%), visual impairment (55.8%) and headache (36.3%), with hypopituitarism as the most common endocrinopathy [13, 14].

In general, pituicytomas have few distinctive imaging characteristics and are often misdiagnosed as pituitary adenoma, meningioma, craniopharyngioma, and other potential lesions in the hypothalamic-pituitary region. In the current series, the tumors were usually of medium size (median maximum diameter 2.0 cm) and less confined to the purely intrasellar (n = 28; 25%). On CT scans, they generally showed isodense, rarely cystic changes, no calcification. On MRI scans, they usually present typically hypo- or isointense on T1WI (n = 37; 92.5%) and iso- or hyperintense on T2WI (n = 32; 80%), with bright, uniform enhancement after administration of gadolinium.

Moreover, flow voids are low-signal images that represent rapid blood flow within the vessels and are common in tumors with rich vascular pedicles. Unlike adenomas, pituicytomas are highly vascularized. Lefevre et al. reported 8 patients, of whom 4 patients with pituicytoma were observed to have intratumoral flow voids [15]. Nagata et al. reported 8 patients, 6 of whom had intratumoral flow voids, including 4 pituicytomas, 1 granular cell tumor, and 1 spindle cell oncocytoma [16]. In addition, 5 patients (22.7%) were observed to have obvious intratumoral flow voids in our cases. Therefore, we support Law Ye et al.’s contention that the intratumoral flow voids can be used to distinguish pituicytoma from some tumors [17]. However, since tumors of posterior pituitary origin, such as granular cell tumor and spindle cell oncocytoma, have the above similar characteristics, the distinction between the three is still problematic.

In conclusion, the possibility of pituicytoma should be highly considered when there are the following conditions: (1) middle-aged people, visual symptoms; (2) hypopituitarism found in endocrine examination; (3) head CT showed that the tumor was of isodense without calcification; (4) head MRI revealed well-circumscribed, medium-sized globular masses entirely located in the suprasellar region and clearly separated from the pituitary gland; (5) iso- or hypointense on T1WI, iso- or hyperintense on T2WI, and homogeneous enhancement after contrast-enhanced scanning; (6) especially when there was flow void in tumor.

### Treatment

Surgery has always been the mainstay of treatment for pituicytoma management. For the vast majority of patients, GTR is the cure for the disease. However, there is no agreement on the degree of resection and the incidence of complications after TS and OT. Feng et al. believed that OT was associated with greater risk of visual loss or other neurological deficit than TS, while TS was associated with greater likelihood of obtaining a GTR [2]. Salge-Arrieta et al. concluded that among the most frequent complications, those secondary to a transitory/permanent lesion of the pituitary gland or stalk stand out, without any apparent difference observed in regards to the surgical approach [13]. Guerrero-Pérez et al. believed that the OT as might expected was associated with higher risks of visual damage and TS with subtotal resection [18]. Secci et al. argued that the extent of tumor removal was not influenced by the choice of surgical approach (either OT or TS) [19]. For the first time, on the basis of a large series of cases, we confirmed statistically that OT is more likely to have postoperative complications, particularly visual deterioration. Besides, it must be pointed out that in this series, suprasellar lesions were mostly removed by OT, while intrasellar lesions tended to be excised by TS (p < 0.0001). Tumor size was an independent factor for GTR of pituicytoma, not related to surgical method.

In the combined cohort, 22 patients developed progression or recurrence after the initial surgery, and further treatment was available in 14 patients. Three patients received radiotherapy, one of them progressed again; 11 patients underwent surgery, four of them progressed again. The median follow-up time for the 14 patients after the second treatment was 20.5 months (ranging from 4 to 60 months). The value of radiotherapy has not been clarified because of the limited number of patients who received standalone radiotherapy after tumor progression. However, it is worth mentioning that none of the 8 patients with non-GTR + RT progressed, including 6 patients who received radiotherapy after the initial surgery and 2 patients who received radiotherapy after the second surgery after progression. (Fig. 5).

### TTP and risk factors

Based on a large number of case series, we confirmed for the first time that male gender, non-GTR and TS are risk factors for recurrence or progression of pituicytoma. It should be noted that in this series of cases, the GTR rate of TS (57.4%) was higher than that of OT (34.4%), but Kaplan–Meier survival analysis showed that patients with TS appeared to be more prone to regrowth. We speculate that the residual volume of tumor after TS is larger than that after OT in patients with non-GTR, so the patients with TS are more likely to progress. Unfortunately, most of the cases are from the previous literature, the specific residual volume of the tumor is not available, so it is impossible to further analyze. Moreover, Zunarelli et al. reported a patient with recurrence after GTR, whose pathology was atypical cells with high mitotic activity and high cytoproliferative index (Ki-67 = 9%), and proposed the hypothesis that atypical histology in pituicytoma may lead to a poor prognosis in terms of recurrence [20]. Although there was no significant difference in the recurrence rate between the Ki-67 ≥ 3% group and Ki-67 < 3% group in the pooled series, we observed that the Ki-67 index of 2 patients with multiple recurrences from our hospital reached 7%.

### Proposed treatment paradigm

In view of the benign and slow growth characteristics of pituicytoma (none of 115 patients died, EMTTP: 89.3 months), asymptomatic patients can choose watchful waiting, but it should be considered that the risk of incomplete resection will increase with the increase of tumor size. In the pooled cohort, 19 of 54 patients with non-GTR (35.2%) progressed, and 3 of 61 patients with GTR (4.9%) relapsed (Fig. 5a). This suggests that GTR of the tumor can achieve a good prognosis. However, features such as abundant blood supply and close adherence to surrounding structures make GTR a challenge. For these tumors, aggressive surgical resection will bear the risk of persistent deterioration of pituitary function. One of our patients underwent a 12-h operation (intraoperative blood loss of 12000 ml) and achieved a near-total resection of the tumor, but the patient remained in a coma for a long time after the operation and was hospitalized in intensive care unit for 48 days. The patient lost sight in the right eye after surgery, accompanied by permanent panhypopituitarism, DI, cognitive impairment and central hyperthermia. Therefore, we agree with Islamian AP et al. that subtotal resection may be preferred to gross aggressive resection in those cases, where the tumour is involving the pituitary stalk, hypothalamus and has invaded surrounding structures [21].

Although the value of radiotherapy after non-GRT is uncertain, we observed no progress in 8 patients (4 male) who received radiotherapy immediately after non-GRT (median follow-up time: 36 months; range: 12–96 months), including 2 male patients who experienced non-GRT + RT after recurrence. Among the 11 patients who underwent reoperation due to tumor recurrence or progression, none of the patients who received GTR after the second operation recurred, while 4 of the 6 patients who did not receive GTR developed again (Fig. 5). Combined with our analysis of the risk factors for tumor recurrence or progression, we suggest that male patients with high Ki-67 index should receive radiotherapy for residual tumor after non-GTR, while the rest of the patients can choose to have regular reexamination. When the tumor progresses or recurs, we recommend a second operation and GTR as far as possible; If GTR is not possible, postoperative radiotherapy for residual tumor is recommended.

### Limitations of the present study

Our findings should be understood taking into account the following limitations. Firstly, the review of the previous literature is limited to the English language. Secondly, due to the rarity of pituicytoma and the lack of randomized data, there may be potential bias (selection bias) in the pooled data analysis. Lastly, the previously published literature covers a lengthy time span, with authors in different specialties reporting cases with varying emphasis, resulting in a lack of information on clinical manifestations, hormonal studies, radiological findings, surgical approaches, and postoperative complications in some cases.

## Conclusions

Given the benign and slow-growing characteristics of pituicytomas, asymptomatic patients may choose a wait-and-watch approach, but the risk of incomplete resection should be considered as the tumor size increases. For symptomatic patients, if tolerable, GTR is advocated as the optimal treatment for pituicytomas. If severe deterioration of pituitary function will be caused after GTR, partial resection of the lesions is recommended. As for residual tumors, male patients with high Ki-67 index should be treated with radiotherapy, while the remaining patients may choose to have periodic review. When the tumor relapses or regrowth, we recommend reoperation and GTR as far as possible; if GTR is not feasible, postoperative radiotherapy is recommended for residual tumor.

## Supplementary Information

Below is the link to the electronic supplementary material.

Supplementary file1 (PDF 28 KB)

Supplementary file2 (PDF 26 KB)

Supplementary file3 (PDF 77 KB)

Supplementary file4 (DOCX 25 KB)

## Data Availability

The datasets generated during and/or analysed during the current study are available from the corresponding author on reasonable request.
