# Supplementary material for: Treatment and prognostic factors of pituicytoma: a single-center experience and comprehensive literature review
Source: Pituitary. 2021 May 12;24(5):754–67. doi: 10.1007/s11102-021-01152-5 (PMC8416853; doi:10.1007/s11102-021-01152-5)
Supplement: Supplementary file 1 — Supplementary file1 (PDF 28 KB) [file 11102_2021_1152_MOESM1_ESM.pdf]

Supplementary material for:

Title: Treatment and prognostic factors of Pituicytoma: a single-center experience and comprehensive literature review

Author: Liu-Dong Wei, MD,[1#] Chao Li, MD,[1#] Da Li, MD,[1] Xing-Ju Liu, MD,[1] Run-Ting Li, MD,[1] Lian-Wang Li,MD,[1] Jun-Mei Wang, MD, PhD,[2] Da-Biao Zhou, MD, PhD,[1]

Corresponding author:

Dr. Da-Biao Zhou. Department of Neurosurgery, Beijing Tiantan Hospital, Capital Medical University. Email:zhoudabiao@bjtth.org. Tel +86-10-59978431. Fax+86-10-59978432.

Dr. Jun-Mei Wang. Department of Neuropathology, Beijing Neurosurgical Institute. Email:wwwjjjmmm1180@sina.com. Tel/ Fax +86-10-59976757.

Journal: Pituitary

Supplementary Table 1. Clinical data of 22 patients with pituicytomas from our hospital.

Case	Sex/age	Presentation; duration(mo)	Endocrinal abnormality	Diameter(mm); consistency/ location	Prediagnosis	Surgical methods/ treatment	Follow-up (mo) /progression
1;1st(§)	M/51	VS;3	LH↓, FSH↓, P4↓	NA	NA	TS/Non-GTR	12/Y
1;2nd(§)	M/52	Null	Null	NA	Null	TS/Non-GTR	12/Y
1;3rd	M/53	Null	Null	25;S/IS+SS	Null	OT/GTR	90/N
2	F/43	DI,VS,Am,Dz; 6	Normal	13;S/SS	CP	OT/GTR	86/N
3	M/32	HA,VS;24	Normal	15;S/SS	CP	OT/GTR	69/N
4;1st(§)	M/47	VS,DL;24	FSH↓,TT4↓	NA	NA	TS/Non-GTR	5/Y
4;2nd	M/47	Null	Null	33;S/IS+SS	Null	OT/Non-GTR	6/ Y
4;3rd	–	–	–	–	–	RT(GKRS)	63/N
5	F/24	VS;1	Normal	23;S/IS+SS	PA	OT/Non-GTR	66/N
6	F/65	Incidental	Normal	30;S+Cys/IS+SS	PA	OT/Non-GTR	66/N
7;1st	M/50	Dz,Vom,VS;2	Normal	15;S/SS	CP	OT/Non-GTR	12/Y
7;2nd	–	–	–	–	–	RT(IMRT;50Gy)	51/N
8	M/48	Dz,HA,VS,DL ;18	LH↓	22;S/IS+SS	MA	OT/Non-GTR+RT (IMRT;56Gy)	62/N
9;1st	M/61	VS;3	TT4 ↓,FT4↓	34;S/IS+SS	PA	TS/Non-GTR	7/Y
9;2nd	M/61	Null	Null	35;S/IS+SS	Null	OT/Non-GTR	16/Y
9;3rd	M/63	Null	Null	32;S/IS+SS	Null	TS/Non-GTR+RT (IMRT;50.4 Gy)	38/N
10	F/60	VS,HA,Dz;24	TT4 ↓, PRL ↑.	24;S/SS	CP	OT/GTR	52/N
11;1st	F/50	VS,HA,Dz;36	Normal	20;S+Cys/IS+SS	PA	TS/Non-GTR	12/Y
11;2nd	F/51	Null	Null	22;S+Cys/IS+SS	Null	OT/Non-GTR	27/N
12	F/50	VS;2	PRL↓	25;S/IS	PA	TS/Non-GTR	38/N
13	F/47	HA;60	Normal	20;S/IS	HP	TS/Non-GTR	37/N

14	F/46	Incidental	Normal	30;S/SS	GCT	OT/GTR	36/N
15	F/38	Incidental	IG1↓,PRL ↑	18;S/SS	PA	OT/Non-GTR	30/N
16	M/55	Dz, Gmastia;9	P4 ↓	26;S+Cys/SS	CP	OT/GTR	25/N
17	M/54	Incidental	LH↓, P4 ↓	23;S/SS	CP	TS/Non-GTR	19/N
18	F/54	HA,Vom,VS;36	ACTH↓,TT3↑, FT3↑, TT4 ↑	20;S/IS	PA	TS/Non-GTR	15/Y
19	M/46	Dz;1	Normal	25;S/SS	MA	OT/Non-GTR	13/N
20	F/35	HA,Dz;5	Normal	20;S/IS+SS	CP	OT/Non-GTR	13/N
21	M/43	W,DL;6	Normal	9;S/IS	PA	TS/GTR	7/N
22	M/52	Dz,HA;1	Normal	30;S/SS	MA	OT/GTR	6/N

ACTH, adrenocorticotropic hormone; Am, amenorrhea; CP, craniopharyngioma; Cys, cystic; DI, diabetes insipidus; DL, decreased libido; Dz, dizziness; F, female; FSH, follicle-stimulating hormone; FT3, free T3; FT4, free T4; GCT, Granular cell tumor; GH, growth hormone; GKRS, gamma knife radiosurgery; Gmastia, gynecomastia; GTR, gross-total resection; HA, headache; HP, Hypophysitis; IGF-1, insulin-like growth factor-1; IMRT, intensity-modulated radiotherapy; IS, intrasellar; LH, luteinizing hormone; M, male; MA, meningioma; mo, month; mm, millimeter; N, no; NA, not available; OT, open transcranial; S, solid; SS, suprasellar; TS, transsphenoidal surgery; TSH, thyroid-stimulating hormone; TT3, total triiodothyronine; TT4, total thyroxine; P4, progesterone; PA, Pituitary adenoma; PRL, prolactin; Vom, vomiting; VS, visual symptoms; W, weakness; Y, yes; 1st, first operation; 2nd, second operation; 3rd, third operation; ↑, Higher than normal upper limit; ↓, Lower than normal lower limit.

(§) Note: The first two operations of Case 1 and the first operation of Case 4 were performed in other hospitals. By inquiring patients,

consulting medical records and comparing head MRI before and after surgery, the chief complaint and surgery data of the patients at the first visit were obtained.
