# Supplementary material for: Treatment and prognostic factors of pituicytoma: a single-center experience and comprehensive literature review
Source: Pituitary. 2021 May 12;24(5):754–67. doi: 10.1007/s11102-021-01152-5 (PMC8416853; doi:10.1007/s11102-021-01152-5)
Supplement: Supplementary file 2 — Supplementary file2 (PDF 26 KB) [file 11102_2021_1152_MOESM2_ESM.pdf]

Supplementary material for:

Title: Treatment and prognostic factors of Pituicytoma: a single-center experience and comprehensive literature review

Author: Liu-Dong Wei, MD,[1#] Chao Li, MD,[1#] Da Li, MD,[1] Xing-Ju Liu, MD,[1] Run-Ting Li, MD,[1] Lian-Wang Li,MD,[1] Jun-Mei Wang, MD, PhD,[2] Da-Biao Zhou, MD, PhD,[1]

Corresponding author:

Dr. Da-Biao Zhou. Department of Neurosurgery, Beijing Tiantan Hospital, Capital Medical University. Email:zhoudabiao@bjtth.org. Tel +86-10-59978431. Fax+86-10-59978432.

Dr. Jun-Mei Wang. Department of Neuropathology, Beijing Neurosurgical Institute. Email:wwwjjjmmm1180@sina.com. Tel/ Fax +86-10-59976757.

Journal: Pituitary

Supplementary Table 2. Clinical data of 22 patients with pituicytomas from our hospital.

Cases	Surgical findings				Immunohistochemical results							Complications
	Texture	Color	Vascu larity	BL (ml)	TTF1	EMA	S100	GFAP	Vim	Syn	ki67 (%)	
1;1st	NA	NA	NA	NA	NA	NA	NA	NA	NA	NA	NA	NA
1;2nd	NA	NA	NA	NA	NA	NA	NA	NA	NA	NA	NA	NA
1;3rd	Fibrous	G-R	Abu	100	NA	-	+	-	+	-	2	HypoTh, HypoCor, VS
2	Soft	G-W	Mod	100	NA	NA	+	+	+	-	4	DI
3	Fibrous	G-R	Ext	100	NA	-	+	-	+	-	1	DL,DI, panHypoPi
4;1st	NA	NA	NA	NA	NA	NA	NA	NA	NA	NA	NA	NA
4;2nd	Soft	Red	Ext	1000	+	±	+	-	NA	±	7	DI,VS,DL, HypoTh, HypoCor
5	Hard	G-Y	Mod	200	NA	NA	+	+	+	NA	NA	PNM, HypoTh, VS
6	Soft	G-Y	Abu	100	NA	-	+	±	+	-	1	DI, HypoTh, VS
7	Fibrous	G-Y	Mild	700	NA	-	+	+	NA	-	NA	HypoGn, PC
8	Soft	G-R	Abu	400	+	-	+	+	+	-	1	DI,VS,DL,PC, HypoGn
9;1st	NA	NA	NA	NA	+	NA	+	-	+	+	NA	HypoTh, DI
9;2nd	NA	NA	NA	NA	+	+	+	-	+	+	7	HypoTh, HypoCor, DI
9;3rd	Soft- fibrous	G-Y	Abu	800	NA	NA	NA	NA	NA	NA	NA	DI, HypoTh
10	Soft	G-R	Abu	600	+	NA	+	+	NA	+	NA	CF,VS, HypoTh, HypoCor, DI
11;1st	Fibrous	G-W	Mod	500	NA	NA	+	+	+	NA	1	CSF leak, HypoTh

11;2nd	NA	NA	NA	NA	+	NA	+	+	NA	NA	5	HypoTh, HypoCor, DI
12	Fibrous	G-W	Mod	100	NA	+	+	+	NA	-	15	HypoTh
13	Fibrous	G-R	Abu	300	+	±	+	+	NA	NA	1	CSF leak, PNM, CF, HypoTh, HypoCor
14	Fibrous	G-R	Mild	100	+	+	+	+	NA	NA	1	No
15	Fibrous	G-R	Ext	700	+	-	+	+	NA	NA	4	HypoTh, HypoCor, HyperPrl, DI,VS
16	Soft- fibrous	G-R	Ext	200	NA	NA	+	±	NA	-	1	HypoGn, HypoTh DI, DL, PC
17	Soft	G-Y	Mod	150	NA	-	+	±	NA	NA	3	PanHypoPi
18	Soft	Red	Abu	200	+	±	+	-	NA	NA	3	HypoTh, HypoCor
19	Fibrous	Red	Ext	1200	+	±	±	-	NA	NA	2	Cog, PC, CF, VS, DI, panHypoPi
20	Soft- fibrous	G-R	Ext	500	+	+	±	+	NA	-	10	DI,PNM,VS, HypoTh, HypoCor
21	Soft	G-W	Mild	100	+	NA	+	±	NA	+	1	HypoTh, HypoCor, VS,DL, DI,
22	Soft	G-B	Abu	400	+	-	+	±	NA	NA	8	DI, PNM, PC, DVT,VS, HypoCor

Abu, abundant; BL, blood Loss; CF, central fever; Cog, cognitive disturbances; CSF, Cerebrospinal fluid; DI, diabetes insipidus; DL, decreased libido; DVT, deep vein thrombosis; EMA, epithelial membrane antigen; Ext, extremely rich; GFAP, glial fibrillary acidic protein; G-B, grayish-brown; G-R, grayish-red; G-W, grayish-white; G-Y, grayish-yellow; HyperPrl, hyperprolactinemia; HypoCor, hypocortisolism; HypoGn, hypogonadism; HypoPi, hypopituitarism; HypoTh, hypothyroidism; Mod, moderate; ml, milliliter; PC, personality change; PNM, post-neurosurgical meningitis; Post, postoperative; Pre, preoperative; Syn, Synaptophy; TTF-1, thyroid transcription factor-1; Vim, vimentin; VS, visual symptoms; (-), negative; (+), positive; (±), focal positive.
