# Supplementary material for: Treatment and prognostic factors of pituicytoma: a single-center experience and comprehensive literature review
Source: Pituitary. 2021 May 12;24(5):754–67. doi: 10.1007/s11102-021-01152-5 (PMC8416853; doi:10.1007/s11102-021-01152-5)
Supplement: Supplementary file 3 — Supplementary file3 (PDF 77 KB) [file 11102_2021_1152_MOESM3_ESM.pdf]

Supplementary material for:

Title: Treatment and prognostic factors of Pituicytoma: a single-center experience and comprehensive literature review

Author: Liu-Dong Wei, MD,[1#] Chao Li, MD,[1#] Da Li, MD,[1] Xing-Ju Liu, MD,[1] Run-Ting Li, MD,[1] Lian-Wang Li,MD,[1] Jun-Mei Wang, MD, PhD,[2] Da-Biao Zhou, MD, PhD,[1]

Corresponding author:

Dr. Da-Biao Zhou. Department of Neurosurgery, Beijing Tiantan Hospital, Capital Medical University. Email:zhoudabiao@bjtth.org. Tel +86-10-59978431. Fax+86-10-59978432.

Dr. Jun-Mei Wang. Department of Neuropathology, Beijing Neurosurgical Institute. Email:wwwjjjmmm1180@sina.com. Tel/ Fax +86-10-59976757.

Journal: Pituitary

Supplementary Table 3. Literature review of published cases of pituicytomas.

Author/year	Sex/age (Years)	Presentation	Radiolical findings(mm)	Treatment	Immunohistochemistry	Complications	F-U(m)/REC
Hurley et al.,1994[22]	F/26	HA,VS	22;S/IS+SS	TS/Non-GTR+RT(50.4Gy)	EMA(-),S100(+),GFAP(+),Vim(+)	DI, HypoCor	36/No
Brat et al.,2000[11]	M/39	HA	NA; S+Cys/IS	TS/GTR	NA	NA	24/No
	M/48	HypoGn	20;S/SS	OT/Non-GTR	NA	NA	8/No
	F/55	VS	NA;S/SS	NA/GTR	S100(+),Vim(+)	NA	13/No
	M/30	HA	NA;S/SS	NA/GTR	S100(+),Vim(+)	NA	14/No
	M/42	VS,DL; HypoGn	NA;S/SS	NA/Non-GTR	S100(+),Vim(+)	NA	15/Yes
	M/46	HypoPi	NA;S/IS+SS	NA/GTR	S100(+),Vim(+)	NA	99/No
	F/83	HA,VS	NA;S/SS	NA/GTR	S100(+),Vim(+)	NA	26/No
First operation	M/42	W,VS; HypoPi	NA;S/IS	TS/Non-GTR	NA	NA	26/Yes
Second operation	M/44	NA	NA	NA/Non-GTR	NA	NA	26/No
Schultz et al.,2001[23]	M/66	W,VS,HA	15;S/IS	TS/GTR	EMA (-), S100 (+), GFAP (-); ki-67(1.5%)	NA	26/No
Figarella-Branger/2002[24]	M/59	W,DL; HypoPi	NA;S/IS	TS/Non-GTR	NA	No	132/No
	M/46	DL,W, Gmastia, VS;	NA;S/SS	NA/GTR	EMA(-),S100(+),GFAP(-),Vim(+),Syn(-)	Hemiparesia	48/No

		HypoGn					
	M/58	W,DL,DI,VS; panHypoPi	NA;S/SS	NA/GTR	EMA(-),S100(+),GFAP(-),Vim(+),Syn(-)	DI, panHypoPi	24/No
Ulm et al.,2004[25]; First operation	M/26	DL,VS; HypoPi	NA;S/IS+SS	TS/Non-GTR	EMA(-),S100(+),GFAP(±),Vim(+),Syn(-);ki-67(2%)	NA	60/Yes
Second operation	M/31	NA	NA;NA/IS+SS	OT/Non-GTR	NA	NA	24/Yes
Kowalski et al.,2004[26]	M/52	Gmastia, DL; panHypoPi	30;S/IS+SS	TS/Non-GTR	S100 (+), GFAP (±); ki-67(1.1%)	No	11/Yes
Shah et al.,2005[27]	F/36	HA, Am	NA;S+Cys/IS+SS	TS/Non-GTR	NA	NA	60/Yes
Nakasu et al.,2006[28]	F/42	Am, Dz	NA;S/IS+SS	OT/Non-GTR	EMA(±),S100(+),GFAP(+)	No	56/No
	F/62	HA,W	NA;NA/SS	OT/Non-GTR	EMA (-), S100 (+), GFAP (+); ki-67(1%)	DI, HypoTh, HypoCor	18/No
Wolfe et al.,2008[29]	F/71	VS	41;S/IS+SS	OT/Non-GTR	EMA(-),S100(+),GFAP(±),Vim(-)	No	18/No
Zhi et al.,2009[30]	F/46	HA, Dz	NA;S/IS	TS/Non-GTR	EMA (+), S100 (+), GFAP (±), Vim (+); ki-67(1%)	No	6/No
	F/45	VS,HA, Dz	NA;S/SS	OT/Non-GTR	EMA (±), S100 (+), GFAP (+), Vim (+); ki-67(5%)	No	3/No
Furtado et al.,2010[31]	F/23	HA,VS, seizure	NA;S/IS	OT/Non-GTR	EMA(-),S100(+),GFAP(±)	DI,HypoTh,3rd cranial nerve palsy	84/No
	F/45	HA,W, Dz	NA;S+Cys/IS	TS/GTR	EMA(-),S100(+),GFAP(+)	No	7/No

Zhang et al.,2010[32]	M/53	VS,DL; HypoGn	20;S/SS	OT/Non-GTR+RT(GKRS;center:24Gy; margin:12Gy)	EMA (-), S100 (+), GFAP (±), Vim (+); ki-67(1%)	VS,DL	13/No
Phillips et al.,2010[33]	F/48	Weight loss, W	2;S/IS	TS/Non-GTR	S100(+),GFAP(-),Vim(+)	NA	4/No
	M/54	DI; panHypoPi	NA;NA/IS+SS	NA/Non-GTR	S100(+),GFAP(-),Vim(+),Syn(-)	NA	12/Yes
Brandão et al.,2010[34]	M/17	HA,VS; HyperPrl	26;S/IS+SS	TS/Non-GTR	S100(+),GFAP(-),Vim(-)	DI	24/No
Ogiwara et al.,2011[6]	M/59	VS,W ,DI	NA;S/SS	TS/GTR	TTF-1(+), EMA (-), S100 (+), GFAP (-), Vim (+); ki-67(5%)	No	16/No
Mao et al.,2011[35]	F/47	HA, Am	16;S/IS	TS/GTR	NA	NA	18/No
	F/51	VS,DI,HA	9;S/SS	OT/GTR	EMA (+), S100 (+), GFAP (±), Vim (+); ki-67(1%)	PanHypoPi ,DI	11/No
Zunarelli et al.,2011[20]; First operation	M/63	HypoPi	15;S/IS	TS/GTR	TTF-1(±), EMA (-), S100 (+), GFAP (±); ki-67(9%)	No	31/Yes
Second operation	M/66	NA	24;NA/NA	TS/Non-GTR+RT	NA	NA	13/No
Secci et al.,2012[19]	M/60	No	NA;S/IS+SS	TS/Non-GTR	NA	No	28/No
	F/35	Am, VS	20;S+Cys/SS	TS/Non-GTR	NA	No	26/No
	F/65	VS	NA;S+Cys/IS+SS	TS/GTR	NA	DI	5/No
Huynh et al.,2012[36]	F/43	VS	19;S/IS+SS	OT/Non-GTR	S100(±),GFAP(+)	HypoTh, HyperPrl	10/No

Kosuge et al.,2012[3]; First operation	M/52	VS,DL; HypoGn	20;S/IS+SS	TS/Non-GTR	S100 (+), GFAP (-); ki-67(2%)	HypoTh, HypoCor	84/Yes
Second operation	M/59	NA	NA	OT/GTR	NA	DI	12/No
Pirayesh et al.,2012[21]	F/51	HA,VS;HypoGn, HyperPrl	19;S/SS	OT/Non-GTR	EMA (+), S100 (+), GFAP (+), Vim (+); ki-67(1%)	PanHypoPi	4/No
Chakraborti et al.,2013[37]	M/20	HA, Vom, VS	NA;S/IS+SS	TS/GTR	EMA (+), S100 (+), GFAP (±), Vim (-); ki-67(0.5%)	No	6/No
	F/7	VS,HA	30;S/SS	TS/GTR	EMA (±), GFAP (+); ki-67(2%)	No	12/No
	M/24	CS ;HyperCor	6;S/IS	TS/GTR	EMA (±), S100 (+), GFAP (±), Vim (+); ki-67(2%)	No	12/No
Tian et al.,2013[38]	M/13	VS,HA	55;S/SS	OT/Non-GTR	EMA(-),S100(+),GFAP(+),Vim(+)	No	18/No
Koutourousiou et al.,2013[39]	M/42	VS; HypoPi	20;S/SS	TS/GTR	NA	DI, DVT	10/No
	M/41	HA, VS; HypoPi	18;S/SS	TS/GTR	NA	DI, hydrocephalus	14/No
	F/63	HA,VS	25;S/SS	TS/GTR	NA	DI, HypoPi	8/No
Ida et al.,2013[40]	F/67	Dz,HA,VS; HypoGn, HyperPrl	26;S/IS+SS	OT/GTR	TTF-1(+), EMA (-), S100 (+), GFAP (±), Syn (-); ki-67(5%)	NA	14/No
Feng et al.,2014[2]	M/61	VS; panHypoPi	38;S+Cys/IS+SS	TS/GTR	EMA (-), S100 (+), GFAP (+), Syn (-); ki-67(1%)	DI, panHypoPi	6/No
	M/37	HypoGn	19;S/SS	TS/GTR	EMA(-),S100(+),GFAP(±),Syn(-)	Diplopy, CSF leak, DI,	56/Yes

						panHypoPi	
Zygourakis et al.,2015[41]	M/58	HA, W, DL; HypoGn	30;S+Cys/SS	OT/GTR	EMA(-),S100(+),GFAP(+)	VS, panHypoPi	36/No
	F/33	HA, G, Am	5;NA/IS	TS/GTR	S100(+),GFAP(+),Vim(+)	HypoTh	24/No
	M/71	DL; panHypoPi	20;S/IS+SS	TS/GTR	TTF-1(+),,S100(+),Vim(+)	PanHypoPi	72/Yes；RT/NA
	F/48	VS,HA	20;NA/IS	NA/Non-GTR	NA	DI, panHypoPi	84/Yes
	M/58	VS,W; panHypoPi	30;NA/IS+SS	NA/GTR	S100(+),GFAP(+),Vim(+)	NA	60/No
Teti et al.,2015[42]	M/36	DL, DI; HyperPrl, HypoGn, HypoTh	NA;S/SS	OT/Non-GTR	S100 (+), GFAP (±), Vim (+); ki-67(3%)	Seizures, subdural empyema, DI, panHypoPi, HyperPrl	36/Yes
Neidert et al.,2016[43]	F/67	VS, HA	25;S+Cys/IS+SS	TS/ GTR	TTF-1(+), EMA (±), S100 (+), GFAP (+); ki-67(6%)	No	3/No
Guo et al.,2016[44]	F/46	CS,HA; HyperCor	NA;S/IS	TS/Non-GTR+RT(56Gy)	EMA (-), S100 (+), GFAP (+); ki-67(1%)	NA	96/No
Yang et al.,2016[14]	M/41	HA, Dz, VS	37;S+Cys/IS+SS	OT/GTR	EMA (-), S100 (+), GFAP (+), Vim (+); ki-67(2%)	Nausea and Vom, HypoPi	17/No
	F/44	Irregular menstruation	22;S/IS+SS	TS/GTR	S100(-),GFAP(-)	DI	43/No
	F/61	HA, Vom; HypoTh	9;S+Cys/IS	TS/GTR	S100 (+), GFAP (+), Vim (+); ki-67(1%)	No	45/No
Peron et al.,2017[45]; First operation	M/49	NA	NA	OT/Non-GTR	S100 (+), GFAP (-), Syn (-); ki-67(1.5%)	Pituitary dysfunction, VS	60/Yes

Second operation	M/55	NA	NA	NA/GTR	S100(+),GFAP(-),Syn(-)	NA	24/No
Nagata et al.,2018[16] 1st	M/70	VS, HA; HypoPi	NA;NA/IS+SS	TS/Non-GTR	TTF-1(+), EMA (+), S100 (-), GFAP (-), Vim (+); ki-67(10%)	Anterior pituitary function deteriorated,	21/Yes;
2nd				RT			38/ Yes
3rd				Non-GTR+RT			NA
Feng et al.,2018[46]	F/56	Acromegaly; GH↑,IGF-1↑,HyperPrl	18;NA/IS+SS	TS/GTR	TTF-1(+),EMA(+),S100(+),GFAP(+),Vim(+)	No	18/No
	F/29	CS; HyperCor	4;NA/IS	TS/GTR	TTF-1(+),EMA(-),S100(+),GFAP(+)	DI, HypoCor	12/No
Cossu et al.,2018[47]	M/77	W,VS, pituitary apoplexy; HypoGn	NA;NA/IS+SS	TS/GTR	TTF-1(+),EMA(-),S100(+),GFAP(-),Vim(+),Syn(-);ki-67(1%)	HypoPi	15/No
Chang et al.,2018[48]	F/39	HyperPrl	4;NA/IS	TS/GTR	TTF-1(+),EMA(-),S100(+),GFAP(-),Vim(+),Syn(-)	No	24/No
	F/21	Am; HyperPrl	10.8;NA/IS	NA/GTR	TTF-1(+),EMA(-),S100(+),GFAP(-),Vim(+),Syn(±)	NA	48/No
	F/26	VS, unsteady gait; HyperPrl	8;NA/IS	NA/GTR	TTF-1(+),EMA(-),S100(+),GFAP(±),Vim(+),Syn(±)	NA	84/No
	M/46	Acromegaly; GH↑,IGF-1↑	11.9;NA/IS	NA/GTR	TTF-1(+),EMA(-),S100(+),GF	NA	96/No

					AP(+),Vim(+),Syn(-)		
	F/53	CS; HyperCor	5.8;NA/IS	NA/GTR	TTF-1(+),EMA(+),S100(+),GFAP(-),Vim(+),Syn(+)	NA	24/No
	F/51	CS; HyperCor	7.6;NA/IS	NA/Non-GTR+RT(SRT;16Gy)	TTF-1(+),EMA(-),S100(+),GFAP(-),Vim(+),Syn(+)	NA	36/No
	F/57	CS; HyperCor	5.1;NA/IS	NA/GTR	TTF-1(+),EMA(-),S100(+),GFAP(-),Vim(+),Syn(+)	NA	24/No
Vellutini et al.,2018[49]	M/61	VS,DI	31;S+Cys/IS+SS	OT/Non-GTR	TTF-1(+),EMA(-),S100(+),GFAP(+),Vim(+),Syn(-);ki-67(1%)	DI,VS	3/No
Lefevre et al.,2018[15]	F/56	CS; HyperCor	NA	TS/GTR	TTF-1(+)	No	3/No
	M/48	VS; HypoTh, HypoGn	NA;NA/SS	OT/GTR	TTF-1(+)	VS,DI	3/No
	M/33	VS; panHypoPi	NA;NA/IS+SS	TS/GTR	TTF-1(+)	CSF leak, DI	12/No
	M/46	VS,HA; panHypoPi	NA;NA/IS+SS	TS/GTR	TTF-1(+)	No	36/No
First operation	F/51	VS,HA,G; panHypoPi	NA;NA/IS+SS	TS/Non-GTR	TTF-1(+)	HypoPi	48/Yes
2nd				RT			36/ No
	F/54	VS,HA;HypoTh, HypoCor	NA;NA/IS+SS	OT/Non-GTR	TTF-1(+)	Hematoma, VS, HypoPi	120/No
First operation	F/48	NA	NA	TS/Non-GTR	NA	NA	24/Yes

Second operation	F/50	VS	NA;NA/IS+SS	TS/GTR	TTF-1(+)	No	60/No
Salge-Arrieta et al.,2019[13]	F/30	VS; HyperPrl	25;S/IS+SS	TS/GTR	TTF-1(+),EMA(-),S100(+),GFAP(±),Vim(+),Syn(-);ki-67(4%)	DI, panHypoPi	16/No
Aki et al.,2019[50]	M/38	DI,DL,VS; HypoGn	NA;S/IS+SS	TS/GTR	TTF-1(+), S100 (+), GFAP (+); ki-67(2%)	DI	10/No
Cossu et al.,2019[51]	M/69	VS; panHypoPi	NA;NA/IS+SS	TS/GTR	TTF-1(+)	PanHypoPi	24/No
	M/21	VS	NA;NA/IS+SS	TS/Non-GTR	TTF-1(+)	PanHypoPi, DI	18/No
Viaene et al.,2019[52]	M/47	Hormonal symptoms	31;NA/IS+SS	NA/Non-GTR	TTF-1(+), EMA (-), S100 (+), GFAP (±), Syn (+); ki-67(3%)	PanHypoPi/DI	100.8/No
	M/64	VS	35;NA/IS+SS	NA/Non-GTR	TTF-1(+), EMA (+), S100 (+), GFAP (-); ki-67(4%)	PanHypoPi/DI	45.6/No
	M/49	Hormonal symptoms	10;NA/SS	NA/Non-GTR	TTF-1(+),EMA(-),GFAP(±),Syn(±)	PanHypoPi/DI	33.6/No
	F/30	VS	17;NA/IS+SS	NA/GTR	TTF-1(+), GFAP (+); ki-67(1%)	PanHypoPi/DI	18/No
	F/48	No	16;NA/SS	NA/GTR	TTF-1(+), EMA (+), S100 (+), GFAP (+); ki-67(1%)	PanHypoPi/DI	16.8/No
	M/76	No	16;NA/SS	NA/GTR	TTF-1(+), EMA (±), S100 (+), GFAP (±); ki-67(3%)	PanHypoPi/DI	6/No

Borg et al.,2020[53]	M/44	HA; HypoTh	NA;NA/IS+SS	TS/GTR	TTF-1(+), S100 (+); ki-67(5%)	NA	24/No
	M/56	DL,W; panHypoPi, HyperPrl	NA;NA/IS+SS	TS/GTR	TTF-1(+); ki-67(5%)	NA	48/No
	F/80	VS; HypoTh, HypoCor	NA;NA/IS+SS	TS/GTR	TTF-1(+), EMA (±), S100 (+); ki-67(5%)	NA	36/No
Marco Pont et al.,2020[54]	F/29	Acromegaly; GH↑,IGF-1↑	17;S/IS	TS/Non-GTR+RT	TTF-1(+)	NA	12/No
McNamara et al.,2020[55]	M/71	No	15;S/IS+SS	TS/GTR	TTF-1(+), S100 (+), GFAP (-), Vim (+), Syn (-); ki-67(3%)	No	24/No

Am, amenorrhea; CS, Cushing's syndrome; CSF, Cerebrospinal fluid; Cys, cystic; DI, diabetes insipidus; DL, decreased libido; DVT, deep vein thrombosis; Dz, dizziness; EMA, epithelial membrane antigen; F, female; F-U follow-up; G, galactorrhea; GFAP, glial fibrillary acidic protein; GH, growth hormone; GKRS, gamma knife radiosurgery; Gmastia, gynecomastia; GTR, gross-total resection; HA, headache; HyperCor, hypercortisolism; HyperPrl, hyperprolactinemia; HypoCor, hypocortisolism; HypoGn, hypogonadism; HypoPi, hypopituitarism; HypoTh, hypothyroidism; IGF-1, insulin-like growth factor-1; IS, intrasellar; M, male; m, months; mm, millimeter; NA, not available; OT, open transcranial; REC recurrence; RT, radiotherapy; S, solid; SRT, stereotactic radiosurgery; SS, suprasellar; Syn, Synaptophy; TS, transsphenoidal surgery; TTF-1, thyroid transcription factor-1; Vim, vimentin; Vom, vomiting; VS, visual symptoms; W, weakness; (-), negative; (+), positive; (±), focal positive; ↑, Higher than normal upper limit.
