# Supplementary material for: Treatment and prognostic factors of pituicytoma: a single-center experience and comprehensive literature review
Source: Pituitary. 2021 May 12;24(5):754–67. doi: 10.1007/s11102-021-01152-5 (PMC8416853; doi:10.1007/s11102-021-01152-5)
Supplement: Supplementary file 4 — Supplementary file4 (DOCX 25 KB) [file 11102_2021_1152_MOESM4_ESM.docx]

22. Hurley, T.R., D'Angelo, C.M., Clasen, R.A., Wilkinson, S.B., Passavoy, R.D.: Magnetic resonance imaging and pathological analysis of a pituicytoma: case report. Neurosurgery **35**(2), 314-317; discussion 317 (1994). doi:10.1227/00006123-199408000-00021

23. Schultz, A.B., Brat, D.J., Oyesiku, N.M., Hunter, S.B.: Intrasellar pituicytoma in a patient with other endocrine neoplasms. Archives of Pathology and Laboratory Medicine **125**(4), 527-530 (2001).

24. Figarella-Branger, D., Dufour, H., Fernandez, C., Bouvier-Labit, C., Grisoli, F., Pellissier, J.F.: Pituicytomas, a mis-diagnosed benign tumor of the neurohypophysis: report of three cases. Acta neuropathologica **104**(3), 313-319 (2002). doi:10.1007/s00401-002-0557-1

25. Ulm, A.J., Yachnis, A.T., Brat, D.J., Rhoton, A.L., Jr.: Pituicytoma: report of two cases and clues regarding histogenesis. Neurosurgery **54**(3), 753-757; discussion 757-758 (2004). doi:10.1227/01.neu.0000108983.50966.b7

26. Kowalski, R.J., Prayson, R.A., Mayberg, M.R.: Pituicytoma. Annals of diagnostic pathology **8**(5), 290-294 (2004). doi:10.1016/j.anndiagpath.2004.07.006

27. Shah, B., Lipper, M.H., Laws, E.R., Lopes, M.B., Spellman, M.J., Jr.: Posterior pituitary astrocytoma: a rare tumor of the neurohypophysis: a case report. AJNR. American journal of neuroradiology **26**(7), 1858-1861 (2005).

28. Nakasu, Y., Nakasu, S., Saito, A., Horiguchi, S., Kameya, T.: Pituicytoma. Neurologia medico-chirurgica **46**(3), 152-156 (2006). doi:10.2176/nmc.46.152

29. Wolfe, S.Q., Bruce, J., Morcos, J.J.: Pituicytoma: Case report. Neurosurgery **63**(1), E173-E174 (2008). doi:10.1227/01.NEU.0000335084.93093.C8

30. Zhi, L., Yang, L., Quan, H., Bai-Ning, L.: Pituicytoma presenting with atypical histological features. Pathology **41**(5), 505-509 (2009). doi:10.1080/00313020903041119

31. Furtado, S.V., Ghosal, N., Venkatesh, P.K., Gupta, K., Hegde, A.S.: Diagnostic and clinical implications of pituicytoma. Journal of Clinical Neuroscience **17**(7), 938-943 (2010). doi:10.1016/j.jocn.2009.09.047

32. Zhang, F., Chen, J., You, C.: Pituicytoma: Case report and review of the literature. Neurology India **58**(5), 799-801 (2010). doi:10.4103/0028-3886.72187

33. Phillips, J.J., Misra, A., Feuerstein, B.G., Kunwar, S., Tihan, T.: Pituicytoma: characterization of a unique neoplasm by histology, immunohistochemistry, ultrastructure, and array-based comparative genomic hybridization. Archives of pathology & laboratory medicine **134**(7), 1063-1069 (2010). doi:10.1043/2009-0167-cr.1

34. Brandão, R.A., Braga, M.H., de Souza, A.A., Reis, B.L., Faraj de Lima, F.B.: Pituicytoma. Surgical neurology international **1**, 79 (2010). doi:10.4103/2152-7806.73802

35. Mao, Z., Xiao, W., Wang, H., Li, Z., Huang, Q., He, D., Zhu, Y.: Pituicytoma: Report of two cases. Oncology letters **2**(1), 37-41 (2011). doi:10.3892/ol.2010.209

36. Huynh, N., Stemmer-Rachamimov, A.O., Swearingen, B., Cestari, D.M.: Decreased vision and junctional scotoma from pituicytoma. Case reports in ophthalmology **3**(2), 190-195 (2012). doi:10.1159/000339242

37. Chakraborti, S., Mahadevan, A., Govindan, A., Sridhar, K., Mohan, N.V., Satish, I.R., Rudrappa, S., Mangshetty, S., Shankar, S.K.: Pituicytoma: report of three cases with review of literature. Pathology, research and practice **209**(1), 52-58 (2013). doi:10.1016/j.prp.2012.10.006

38. Tian, Y., Yue, S., Jia, G., Zhang, Y.: Childhood giant pituicytoma: A report and review of the literature. Clinical neurology and neurosurgery **115**(10), 1943-1950 (2013). doi:10.1016/j.clineuro.2013.07.032

39. Koutourousiou, M., Gardner, P.A., Kofler, J.K., Fernandez-Miranda, J.C., Snyderman, C.H., Lunsford, L.D.: Rare infundibular tumors: Clinical presentation, imaging findings, and the role of endoscopic endonasal surgery in their management. Journal of Neurological Surgery, Part B: Skull Base **74**(1), 1-11 (2013). doi:10.1055/s-0032-1329619

40. Ida, C.M., Yan, X., Jentoft, M.E., Kip, N.S., Scheithauer, B.W., Morris, J.M., Dogan, A., Parisi, J.E., Kovacs, K.: Pituicytoma with gelsolin amyloid deposition. Endocrine pathology **24**(3), 149-155 (2013). doi:10.1007/s12022-013-9254-y

41. Zygourakis, C.C., Rolston, J.D., Lee, H.S., Partow, C., Kunwar, S., Aghi, M.K.: Pituicytomas and spindle cell oncocytomas: modern case series from the University of California, San Francisco. Pituitary **18**(1), 150-158 (2015). doi:10.1007/s11102-014-0568-7

42. Teti, C., Castelletti, L., Allegretti, L., Talco, M., Zona, G., Minuto, F., Boschetti, M., Ferone, D.: Pituitary image: pituicytoma. Pituitary **18**(5), 592-597 (2015). doi:10.1007/s11102-014-0612-7

43. Neidert, M.C., Leske, H., Burkhardt, J.K., Kollias, S.S., Capper, D., Schrimpf, D., Regli, L., Rushing, E.J.: Synchronous pituitary adenoma and pituicytoma. Human pathology **47**(1), 138-143 (2016). doi:10.1016/j.humpath.2015.08.017

44. Guo, X., Fu, H., Kong, X., Gao, L., Wang, W., Ma, W., Yao, Y., Wang, R., Xing, B.: Pituicytoma coexisting with corticotroph hyperplasia: Literature review with one case report. Medicine (United States) **95**(10) (2016). doi:10.1097/MD.0000000000003062

45. Peron, S., Mandelli, J., Galante, N., Colombo, S., Locatelli, D.: Recurrent Pituicytoma with Pseudoaneurysm: Report of a Challenging Case. World neurosurgery **105**, 1043.e1041-1043.e1045 (2017). doi:10.1016/j.wneu.2017.06.171

46. Feng, Z., Mao, Z., Wang, Z., Liao, B., Zhu, Y., Wang, H.: Non-adenomatous pituitary tumours mimicking functioning pituitary adenomas. British journal of neurosurgery, 1-5 (2018). doi:10.1080/02688697.2018.1464121

47. Cossu, G., Dimitriou, J., Brouland, J.P., Daniel, R.T., Messerer, M.: An exceptional presentation of pituicytoma apoplexy: A case report. Oncology letters **16**(1), 643-647 (2018). doi:10.3892/ol.2018.8625

48. Chang, T.W., Lee, C.Y., Jung, S.M., Lai, H.Y., Chen, C.T., Yeap, M.C., Chuang, C.C., Hsu, P.W., Chang, C.N., Tu, P.H., Lee, S.T.: Correlations between clinical hormone change and pathological features of pituicytoma. British journal of neurosurgery **32**(5), 501-508 (2018). doi:10.1080/02688697.2018.1472212

49. Vellutini, E.A.S., Becker, P.H.P., Godoy, L.F., Guerreiro, N.F.C., Mattedi, R.L., de de Oliveira, M.F.: Epithelioid pituicytoma: An unusual case report. Surgical neurology international **9**, 145 (2018). doi:10.4103/sni.sni_319_17

50. Aki, T., Inoue, A., Kohno, S., Nishida, N., Yamashita, S., Fukushima, M., Matsumoto, S., Suehiro, S., Nishikawa, M., Ozaki, S., Shigekawa, S., Watanabe, H., Kitazawa, R., Kunieda, T.: Clinical features and endoscopic findings of pituicytoma in the sellar region: A case report and review of the literature. Interdisciplinary Neurosurgery: Advanced Techniques and Case Management **16**, 58-61 (2019). doi:10.1016/j.inat.2018.12.013

51. Cossu, G., Brouland, J.P., La Rosa, S., Camponovo, C., Viaroli, E., Daniel, R.T., Messerer, M.: Comprehensive Evaluation of Rare Pituitary Lesions: A Single Tertiary Care Pituitary Center Experience and Review of the Literature. Endocrine pathology **30**(3), 219-236 (2019). doi:10.1007/s12022-019-09581-6

52. Viaene, A.N., Lee, E.B., Rosenbaum, J.N., Nasrallah, I.M., Nasrallah, M.P.: Histologic, immunohistochemical, and molecular features of pituicytomas and atypical pituicytomas. Acta neuropathologica communications **7**(1), 69 (2019). doi:10.1186/s40478-019-0722-6

53. Borg, A., Jaunmuktane, Z., Dorward, N.: Tumors of the Neurohypophysis: One Unit's Experience and Literature Review. World neurosurgery **134**, e968-e978 (2020). doi:10.1016/j.wneu.2019.11.043

54. Marco del Pont, F., Villalonga, J.F., Ries-Centeno, T., Arakaki, N., Katz, D., Cervio, A.: Pituicytoma Associated with Acromegaly and Cushing Disease. World neurosurgery **136**, 78-82 (2020). doi:10.1016/j.wneu.2019.12.085

55. McNamara, K.J., Shaw, S., Saravanappa, N.: A recurrent case of pituicytoma 16 years later. Annals of the Royal College of Surgeons of England, 1-3 (2020). doi:10.1308/rcsann.2020.0004
